# Intravitreal Transplantation of Retinal Progenitor Cells Improves Outcome Measures in a Rat Model of Diabetic Retinopathy

**DOI:** 10.3390/ijms26199450

**Published:** 2025-09-27

**Authors:** Jing Yang, Geoffrey P. Lewis, Chin-Hui Hsiang, William Cho, Steven Menges, Kaili Ding, Gabriel Luna, Steven K. Fisher, Henry Klassen

**Affiliations:** 1 Gavin Herbert Eye Institute, Sue & Bill Gross Stem Cell Research Center, University of California, Irvine, CA 92697, USA; jyang22@uci.edu (J.Y.);; 2 Neuroscience Research Institute, University of California, Santa Barbara, CA 93106, USA; g_lewis@ucsb.edu (G.P.L.); stevef916@ucsb.edu (S.K.F.)

**Keywords:** stem cells, treatment model, neuroprotection, cytoprotection, vascular disease

## Abstract

Diabetic retinopathy (DR) is a major source of retinal disease and vision loss worldwide. Current treatments fail to address the loss of neurons and are associated with significant side effects. Here, we investigated whether retinal progenitor cells (RPCs) could improve anatomic and functional outcomes in a rat model of DR. Male Long Evans (LE) rats were given streptozotocin (STZ), and the induction of diabetes was confirmed prior to the intravitreal injection of RPCs, either allogeneic (no immunosuppression) or human (with cyclosporin A), at 1 week post-induction. Animals were tested at 6 weeks post-induction via electroretinogram (ERG), optomotor response (OR), and contrast sensitivity (CS). Retinas were harvested post-mortem, 8 weeks post-STZ induction, and analyzed using immunohistochemistry (IHC). In rat RPC-treated eyes, ERG (b-wave, oscillatory potentials), OR, and CS all showed a positive effect for cell treatment versus controls. IHC showed a markedly diminished extravasation of albumin, a decreased VEGF expression, and an improved morphology in cellular and synaptic layers. Human RPC-treated eyes replicated a subset of these results. Together, this provides evidence of both anatomic and functional treatment effects in a rat model of DR, encompassing retinal neuroprotection as well as improved vascular integrity, thereby supporting the further investigation of intravitreal RPCs for the treatment of this condition.

## 1. Introduction

It is estimated that close to 600 million people suffer with diabetes globally [1], with diabetic retinopathy (DR) being the most common eye disease caused by diabetes and a major source of visual loss worldwide. It is a leading cause of blindness in adults [2]. DR can vary in severity, often growing worse over time, and usually affects both eyes. Being common, DR is a major source of visual disability in working-age people. This prevalence further increases with age, and is highest in the 65+ age group, a rapidly increasing demographic segment in industrialized nations.

Like diabetes itself, diabetic retinopathy has long been known to be a disease of blood vessels, and, as the name implies, DR specifically involves the vasculature of the retina, manifesting as a variety of microvascular lesions and resulting in ischemia and the leakage of fluid or blood in and around this delicate light-sensing tissue [2]. Considerable research has gone into identifying the cellular and molecular basis of these changes. Most notably, the expression of the vascular endothelial growth factor (VEGF) by ischemic tissue is known to be important to the pathophysiology of the disease [3], particularly in neovascular complications. Inhibiting this pathway has been an important strategy for the therapeutic management of DR [2,3,4,5].

Less obvious and perhaps under-appreciated is the importance of a neural degenerative component in DR [4,6,7]. While vascular abnormalities may wax and wane, the loss of retinal neurons results in permanent visual defects, often progressing to blindness. No current therapy directly addresses the irreversible component of neuronal cell death within the retina, although the process begins relatively early in the disease [2].

Historically, quite the opposite has been the case, especially prior to anti-VEGF agents. The mainstay of treatment for DR has long been the application of laser burns to the retina in the form of laser photocoagulation. More recently, the advent of injectable anti-VEGF agents has provided an alternative to this destructive approach, particularly for the treatment of neovascular lesions [5], while intraocular corticosteroids have a role in treating DR-associated macular edema [2]. In certain complex or advanced cases, e.g., involving complications of refractory neovascularization such as intravitreal hemorrhage, surgery may be indicated. However, even in combination, these treatments continue to suffer from a limited effectiveness, transient utility, and significant rates of complications [8,9,10]. Crucially, no treatments address the loss of retinal neurons. Providing neuroprotection to the retina in DR can therefore be identified as an unmet medical need [4].

Our team has developed a cell-based therapy with a significant, reproducible neuroprotective benefit in rodent models of retinal degeneration [11,12,13]. This treatment is based on retinal progenitor cells (RPCs) and is currently being evaluated clinically in orphan condition retinitis pigmentosa [14,15,16]. Allogeneic RPCs are injected into the vitreous cavity, in the absence of surgery or immune suppression, with the goal of rescuing and restoring photoreceptor function. Clinical data from our single arm phase 1/2a trial have been consistent with excellent tolerability and provide initial indications of visual benefits [17]. These results raise the interesting possibility that the same approach might also be applicable to DR, a much more common condition.

To investigate the scientific validity of using a cell-based neuroprotective therapy in patients with DR, RPCs were tested in the commonly used streptozotocin (STZ) rat model of DR. This work included the intravitreal injection of rat and human RPCs. Both neuron-related and extra-neuronal endpoints were evaluated. The resulting data indicate that intravitreal RPCs are well tolerated in this model, are not rejected as allografts, and mediate a broad range of positive treatment effects on the STZ retina. These include an improved vascular integrity, a relative normalization of VEGF expression, a preserved morphology of neuronal and non-neuronal retinal cell types, and enhanced electrophysiological and behavioral measures of visual performance versus controls.

## 2. Results

### 2.1. Rat Cell (rRPC) Study

**Figure 1:**
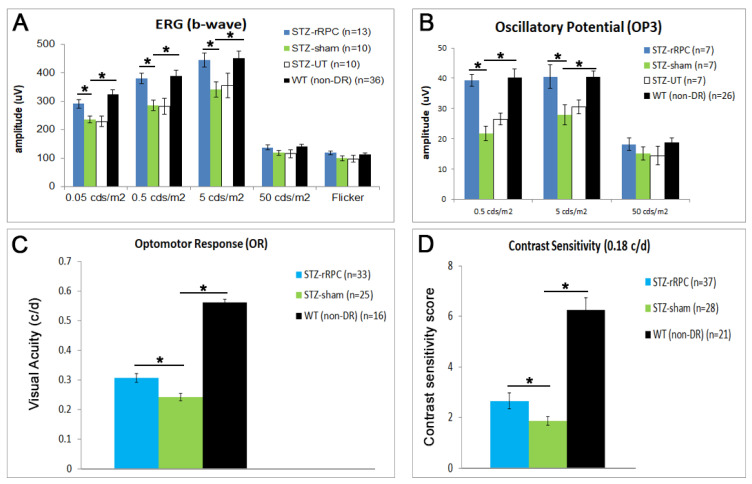
ERG data showing b-wave (**A**) and OP3 peak amplitudes (**B**) for allogeneic cell-treated group vs. sham and untreated controls, in both scotopic (rod-related) and photopic (cone-related) stimulus ranges. Scotopic results show significant improvement for cell vs. sham. Optomotor response (**C**) and contrast sensitivity at 0.18c/d (**D**) show significant improvement for cell vs. sham. The *n*-count refers to number of eyes. * *p* < 0.05 (Student’s *t*-test, unpaired, two-tailed).

This efficacy study included the functional and anatomical assessment of intravitreal allogeneic rat RPC (rRPC) transplantation in the STZ model. Pigmented male Long Evans rats were induced via 75 mg/kg STZ (IP injection) and, 1 week later, after diabetes was confirmed, rRPCs (100k in 2 uL) or a carrier buffer (BSS Plus, sham) were injected into the vitreous cavity (*n* > 20 each arm). Blood glucose was measured weekly for 8 weeks. The functional assessment of electroretinogram (ERG), optomotor response (OR), and contrast sensitivity (CS) was performed at 6w and in each case showed a positive effect for cell treatment (Figure 1). ERG showed a near normalization of response measures, including the amplitude of the b-wave and OP3 oscillatory potential versus the sham and untreated controls in the scotopic range (Figure 1A,B). The a-wave responses did not reach statistical significance. OR and CS results were also significant versus the sham, although clearly diminished versus non-DR (wild-type) animals (Figure 1C,D).

**Figure 2:**
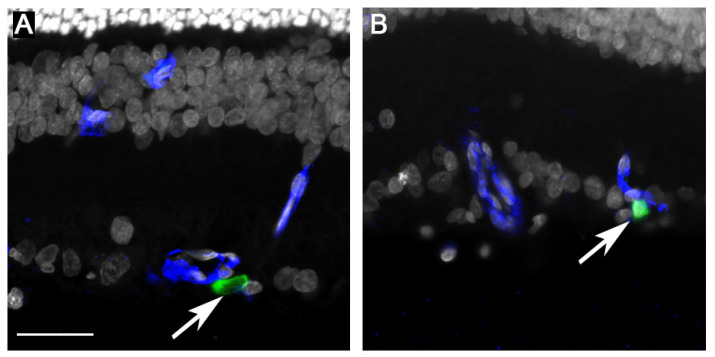
Immunohistochemical analysis of STZ-induced DR retinas after rRPC treatment (STZ-rRPC) (**A**,**B**). Isolectin B4 (blue) labels blood vessels, while E1A (green) is an endogenous reporter that labels donor rRPCs (arrows). Here, we see association of rRPCs with vessels in the inner retina. Representative examples selected from total animal number (*n* = 12). Scale bar = 50 µm (applicable to (**A**,**B**)).

**Figure 3:**
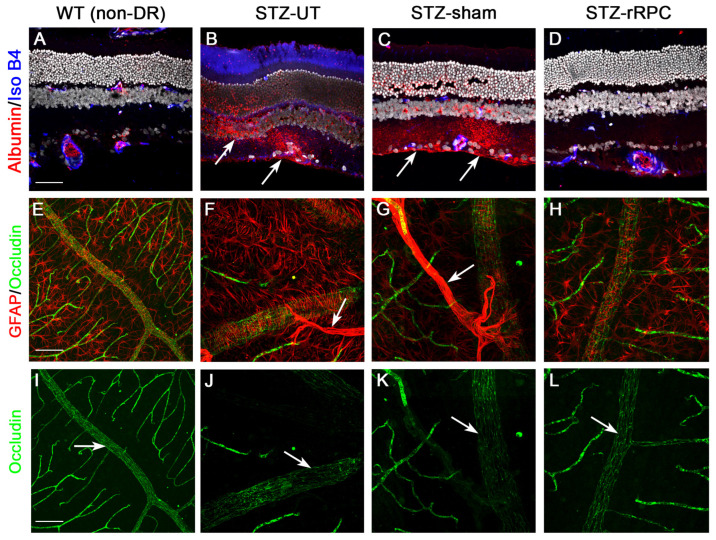
Immunohistochemical analysis of wild-type (WT) and STZ-induced DR retinas (STZ-UT), with comparison to STZ-sham and STZ-rRPC-treated examples. Lack of albumin (red) extravasation (arrows) shows that rRPCs (far right) resist blood retinal barrier breakdown (**A**–**D**). GFAP (red) shows fewer disorganized Muller cells and astrocytes (arrows) in rRPC-treated eyes vs. STZ controls (**E**–**H**). Occludin (green) shows improved vascular tight junctions (arrows) in rRPC-treated vs. STZ controls (**I**–**L**). Representative examples selected from animal groups of number *n* = 7 (WT), 5 (STZ-UT), 10 (STZ-sham), 12 (STZ-rRPC), respectively. Scale bar = 50 µm (applicable throughout, (**A**–**L**)).

**Figure 4:**
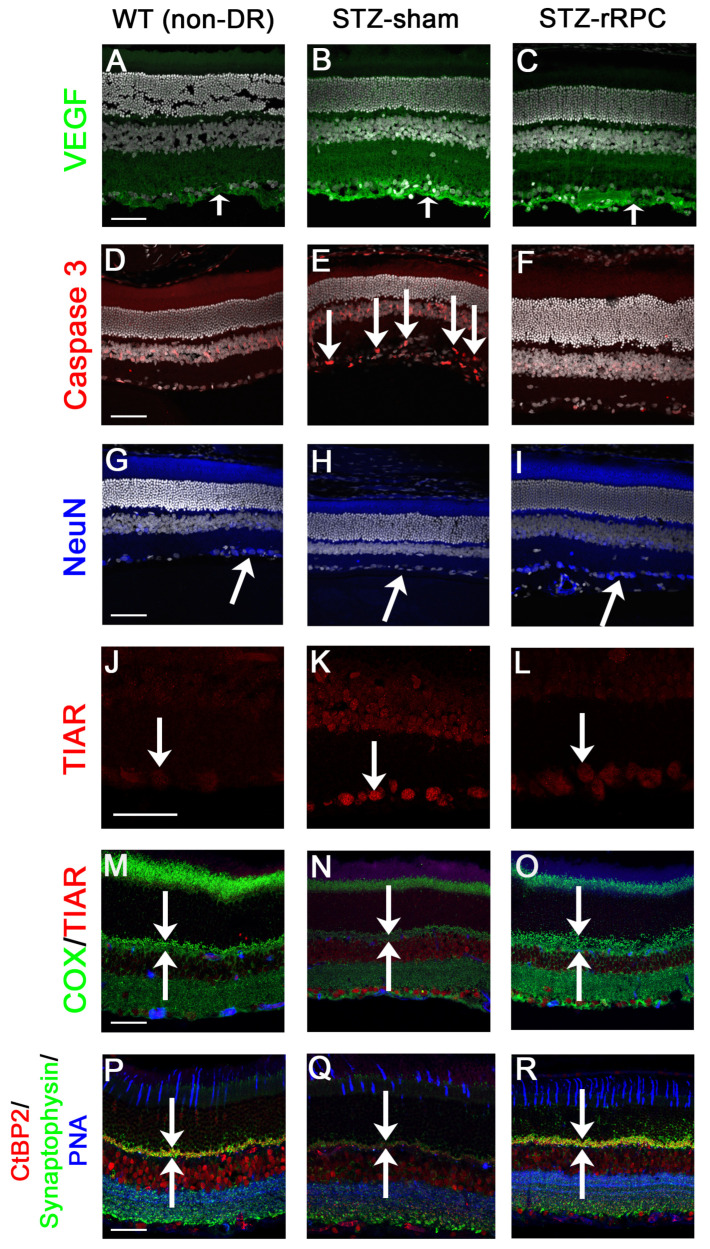
Immunohistochemical analysis of wild-type (WT) and STZ-sham with comparison to STZ-rRPC examples. VEGF (green, arrows) is associated with retinal ischemia and is low in WT (**A**) and elevated in STZ retinas, as seen in STZ-sham (**B**), but less so in rRPC-treated eyes (**C**). Caspase 3 (red) is absent from WT (**D**) but clearly labels apoptotic retinal ganglion cells in STZ retina ((**E**), arrows), while markedly diminished with rRPC treatment (**F**). NeuN (blue) labeling of neurons in the GCL. In STZ-sham eyes, NeuN is decreased (**H**) vs. non-STZ (WT, (**G**)), but not in cell-treated STZ eyes (**I**), indicating neuroprotection. TIAR (red) moves from the nucleus to the cytoplasm into stress granules under stressful conditions. It is elevated in shams (**K**) compared to rRPC-treated eyes (**L**). COX (green) labels mitochondria in the photoreceptor synaptic terminals. COX in the OPL is much stronger in WT (**M**) and rRPC-treated eyes (**O**) vs. shams (**N**), indicating neuroprotection. Synaptophysin (green) labels synaptic vesicles, CtbP2 (red) labels synaptic ribbons, and PNA (blue) labels cones. All are strongly expressed in WT (**P**), diminished in STZ-sham (**Q**), but preserved in rRPC-treated retinas (**R**), indicating neuroprotection. Representative examples selected from animal groups of number *n* = 7 (WT), 10 (STZ-sham), and 12 (STZ-rRPC), respectively. Scale bars = 50 µm throughout (note: magnification is higher for (**J**–**L**), scale bar is longer).

After the 6w post-induction timepoint, a high rate of cataract formation was observed, likely due to hyperglycemia, impeding longer-term functional assessment (cataract score given prior to sacrifice). Immunohistochemical analysis, performed at 8w post-induction, showed improved microvascular findings, decreased intraretinal VEGF, and enhanced neuronal survival in rRPC-treated eyes (Figure 2, Figure 3 and Figure 4).

### 2.2. Human Cell (hRPC) Study

**Figure 5:**
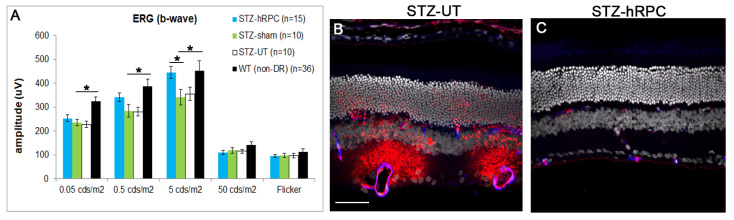
Human RPC xenografts in STZ rats. (**A**) ERG: b-wave amplitude in hRPC-treated (blue) is rescued vs. sham (green) and untreated (white), with WT in black. Significant improvement for cell vs. sham at 5 cds/m^2^. Indicated *n* counts refer to number of eyes. * *p* < 0.05 (Student’s *t*-test, unpaired, two-tailed). (**B**) Diffuse albumin leakage (red) in STZ-UT. (**C**) Lack of albumin extravasation from intact intraretinal vessels in STZ-hRPC-treated animal. Representative examples selected from animal groups numbering *n* = 5 (STZ-UT) and 6 (STZ-hRPC), respectively. Scale bar = 50 µm (applicable to (**B**,**C**)).

The results seen with allogeneic transplantation of rat RPCs (rRPCs) were partially replicated using a human RPC product (Figure 5). Evidence of functional rescue was obtained via ERG for the b-wave amplitude (Figure 5A) but not for the a-wave, oscillatory potentials, OR, or CS, while vascular integrity and neuroprotection were confirmed via the markers used in the rRPC study. Intraretinal VEGF was again decreased for the cell-treated group versus the sham-treated group.

**Figure 6:**
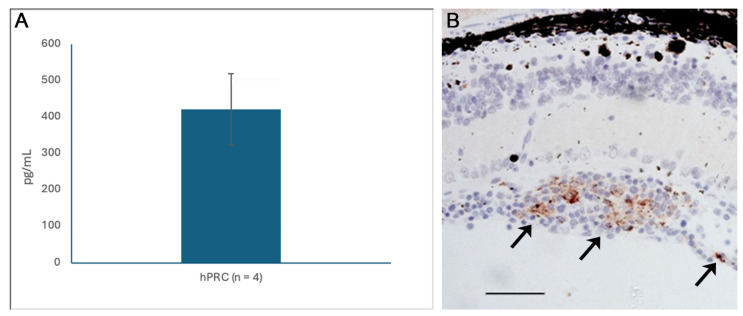
Human RPCs express the antiangiogenic and neuroprotective factor PEDF. Expression of PEDF by hRPCs as demonstrated by ELISA in culture (**A**), and immunocytochemistry (**B**) post-transplantation to the vitreous cavity in STZ-hRPC rats (brown labeling, arrows). Representative example selected from animal group numbering *n* = 6 (STZ-hRPC); scale bar = 100 µm.

In addition, the expression by hRPCs of the pigment epithelium-derived factor (PEDF), reported previously [11], was verified here both in culture using ELISA and in vivo following placement in the vitreous (Figure 6).

## 3. Discussion

Diabetic retinopathy (DR) is a common and potentially debilitating microvascular disease associated with diabetes, for which the available treatments are of limited effectiveness and are themselves associated with significant morbidity. In particular, the need for neuroprotection in the setting of DR has not been addressed. To that end, we have proposed the use of our cell-based RPC therapy as a way of meeting this challenge. Here, we injected RPCs (either allogeneic or human) into STZ-induced diabetic rats, an animal model of DR. These studies show that intravitreal RPCs confer widespread treatment effects that include an improved vascular integrity in association with a decreased intraretinal expression of the key angiogenic factor VEGF, as well as the expression by intravitreal RPCs of the antiangiogenic cytokine PEDF. There was an improved viability of neurons across the retina, along with associated synapses. The mechanism of action appears to involve the expression by hRPCs of a cocktail of secreted factors [11], notably including PEDF, which has a putative neuroprotective role in addition to being antiangiogenic [11]. The rescue was confirmed to be functional and has been partially replicated with human RPCs within the limitations of the xenograft model.

It is widely recognized that allogeneic transplantation generates superior results in comparison to xenografting, and that was the case here. However, this was expected due to the limitations of the model and does not necessarily reflect the potential of the cells being tested. A major factor is immunological, with cells from discordant species eliciting graft rejection. While allogeneic RPCs are remarkably well tolerated in the vitreous cavity [13], human xenografts to the same site typically do require systemic immune suppression, and this can negatively impact functional rescue, including ERG and OR activity in rats [12], thereby artifactually suppressing the functional readout. Moreover, this is likely to include oscillatory potentials (OP), which have relevance in the present study. Beyond immune considerations, other cellular and molecular mismatches between species seem likely, any of which might adversely impact treatment outcomes. Taken together, allogeneic transplantation is preferable whenever optimal graft performance is of interest, including in proof-of-concept discovery research or clinical use. Nevertheless, xenogeneic transplantation continues to have an experimental role, particularly in pre-clinical translational research where a candidate human cell type is evaluated for disease-modifying activity in an animal model. The human cell work presented here was undertaken to extend the rat cell findings to a therapeutic cell type, rather than imply the equivalence of allogeneic versus xenogeneic readouts.

The disease target, diabetic retinopathy (DR), can be seen as a microcirculatory disorder of the retina resulting from the deleterious metabolic effects of hyperglycemia. It has gradually come to be appreciated that an element of retinal neurodegeneration is already present before microcirculatory abnormalities can be detected in routine ophthalmoscopic examinations. Early functional defects include the loss of oscillatory potentials and the b-wave on the electroretinogram (ERG) [18], as well as deficits in contrast sensitivity, dark adaptation, color vision, and visual fields. Diabetes impacts the structure and function of all retinal cell types. Postmortem human retinas from diabetic patients exhibit markers of apoptosis in ganglion cells. Studies in animals confirm the apoptosis of retinal neurons, glial activation, impaired glial cell metabolism, and activation of microglia [19,20]. Therefore, retinal neurodegeneration is an important event occurring early in the pathogenesis of DR, with onset prior to the time of standard clinical diagnosis. Neuronal cell loss both predates and participates in the development of the characteristic microcirculatory abnormalities long associated with the condition [21,22,23]. Because of this, DR falls within the large group of retinal diseases where an intraocular therapy with neuroprotective activity could be of value.

Unmodified RPCs exhibit both neuroprotective and pro-vascular treatment effects, based on the current and previous studies. Factors implicated in RPC-mediated neuroprotection have previously been explored in retinal dystrophic RCS rats and include not only PEDF but the other known neuroprotective factors bFGF and MANF, as well as additional putative neuroprotective factors including osteopontin, pleiotrophin, midkine, and humanin, as described elsewhere [11]. The vascular stabilizing activity of RPCs is, however, a novel finding of particular interest in the setting of DR.

This vascular support might be explained in part by the relatively normalized levels of intraretinal VEGF associated with RPC treatment. In addition, the expression by RPCs of PEDF could further contribute to this positive outcome, since this factor is a naturally occurring inhibitor of VEGF activity. In the retina, the balance between VEGF and PEDF has been seen as a determinant of pathological angiogenic activity [24]. In vitreous diabetic eyes, VEGF is known to be abnormally high, and PEDF is reportedly low [25]. PEDF is not necessarily the direct cause of lower VEGF levels, but is thought to interfere with VEGF receptors and intracellular signaling. The relative normalization of VEGF levels associated with RPC injection is consistent with improved anatomical and physiological outcomes in treated eyes and deserves further investigation.

The positive impact of RPCs on the integrity of the retinal vasculature, as well as neuronal survival and function, is not entirely surprising given the crucial role of these cells during retinal development and vasculogenesis. Whereas the term neuroprotection tends to imply an effect limited to neurons, it is important to recognize that such influences need not be restricted in that way. Indeed, a broader cytoprotective phenomenon appears to be in play here. Again, this should not be entirely surprising since metabolic and chronic degenerative diseases typically involve the mitochondria, where the mediation of cell survival or death pathways generally occurs. Considering the ubiquity and essential role of mitochondria across eukaryotic cells, a degree of overlap can be expected, and natural synergies can be exploited.

We are developing a cell-based therapy for retinal disease which is currently undergoing clinical testing in retinitis pigmentosa (RP), a severe form of retinal degeneration [15,16]. Our phase 1/2a data support the safety and visual benefit of intravitreal RPCs in RP [17], which has been granted a regenerative medicine advanced therapy (RMAT) designation by the FDA, providing further support for the intravitreal RPC approach.

The present study in the STZ rat model provides an initial in vivo validation for our proposal that the neuroprotective effects of intravitreal RPCs may be beneficial in the setting of DR, with improved neurophysiological responses versus controls. In addition, changes in key vascular endpoints were also associated with RPC treatment, including a decrease in both intraretinal VEGF levels and vascular leakage. By impacting neuronal function and vascular integrity, RPC treatment could have potential advantages over the current therapeutic standard. The longer duration of the effect from a cell-based therapy versus protein-based agents is also an advantage, particularly from the perspective of patients who must endure repeated intraocular injections. Furthermore, the anticipated safety profile (based on initial clinical data in RP) could also support this approach versus the current therapeutic standard, particularly long-acting steroids that have major ocular side effects and often require surgery for cataracts and intractable glaucoma. Finally, it should be noted that the use of RPCs does not rule out a combination therapy with the existing modalities, thus providing additional flexibility. This could make future human proof-of-concept trials easier to enroll. The addition of a cytoprotective strategy could be advantageous regardless of other modalities in use.

## 4. Materials and Methods

STZ-induction is a commonly used model of DR, yet there are many variations in terms of dosage, single versus multiple induction, animal strain, route of injection, and potential use of insulin compensation. Progression of DR is also variable depending on animal strain, sex, and functional and histological endpoint assessments [26,27,28]. We therefore conducted two extensive pilot studies in the lead-up to the current effort to select animal stain, optimize STZ dosage, evaluate visual assessments, and determine timepoints for functional and anatomic evaluation.

### 4.1. Animals

Pigmentated male Long Evans (LE) rats purchased from Charles River were used for this study and were housed in a vivarium at the University of California, Irvine, under a 12 h light/dark cycle (maximum 7.7 lux at cage level). Animals were housed and handled in accordance with guidelines set forth by the Institutional Animal Care and Use Committee (IACUC) at the University of California, Irvine.

### 4.2. Induction of Diabetes

Diabetes was induced via intraperitoneal injection of streptozotocin (STZ, Sigma-Aldrich, St. Louis, MO, USA, Cat # S0130), given at 75 mg/kg body weight in a citrate buffer solution. Rats were 8 weeks old at time of STZ induction. Prior to injection, LE rats were fasted overnight (minimum of 16 h). Blood sugar was measured from a prick of the tail vein via hand-held blood glucometer (OneTouch Ultra Mini, Malvern, PA, USA). Following streptozotocin (STZ) administration, serum blood sugar levels and weight were measured weekly, for a total of 8 weeks until sacrifice. These measurements were taken from both the diabetically induced test group and non-diabetic control group. Successful induction of the diabetic model was confirmed by elevated blood glucose > 350 mg/dL (normal range 140 ± 40 mg/dL) and clinical observation of excessive water consumption (polydipsia), excess urination (polyuria), and absence of weight gain after 1 week (1 w).

### 4.3. Treatment Groups

After screening for successful induction via STZ, animals were randomized to a treatment group. In addition to un-induced wild-type (WT, non-DR), the groups were STZ untreated (STZ-UT), STZ sham-treated (STZ-sham), STZ rat RPC-treated (STZ-rRPC), and STZ human RPC-treated (STZ-hRPC). All functional testing was performed with the tester blind to treatment group.

### 4.4. Cell Transplantation

One week after STZ injection, once diabetes was confirmed, rats were anesthetized via intraperitoneal injection of a short-acting combination of ketamine (30 mg/kg, Mylan Institutional, Galway, Ireland, Cat # 26637-411-01) and dexmedetomidine (0.1 mg/kg, Zoetis, Parsippany, NJ, USA, Cat # 122692-5). A 2 µL aqueous solution of either vehicle (balanced salt solution: BSS PLUS, Alcon Laboratories, Fort Worth, TX, USA, Cat # 0065080050) or cell suspension containing either xenogeneic hRPC or allogeneic rRPC was injected into the vitreous cavity, depending on treatment group. After surgery, atipamezole (1.0 mg/kg, Zoetis, Parsippany, NJ, USA, Cat # 107204-6) was given intraperitoneally. Animals were returned to the cage with soft food. No immunosuppressive drugs were used for the allotransplantation group, either treatment or sham. For xenotransplantation, immunosuppression was used for all animals, including the associated sham group, consisting of daily dexamethasone injections (1.6 mg/kg intraperitoneally, West-Ward Pharmaceuticals, Eatontown, NJ, USA, Cat # 0641-0367-21) for 2 weeks from the day of xenotransplantation, as well as cyclosporine-A in the drinking water (210 mg/L, Teva Pharmaceuticals, Parsippany, NJ, USA, Cat # 0172-7313-20) from transplantation until the time of euthanasia. All procedures were conducted in accordance with the ARVO Statement for the Use of Animals in Ophthalmic and Vision Research.

### 4.5. Electroretinography (ERG)

Visual function was evaluated by full-field electroretinography (ERG) using a Ganzfeld stimulator at 6 weeks post-STZ injection. All animals were dark-adapted overnight (>12 h), and all testing was conducted under dim red light. Before the test, each animal’s eye was dilated with one drop each of topical Tropicamide 1% ophthalmic solution (Bausch & Lomb, Rochester, NY, USA, Cat # 24208-585-64) and Phenylephrine 2.5% ophthalmic solution (Akorn Pharmaceuticals, Lake Forest, IL, USA, Cat # 17478-201-15), and animals were then anesthetized by intraperitoneal injection using a long-acting combination of Ketamine 70 mg/kg (Mylan Institutional, Galway, Ireland, Cat # 26637-411-01) and Xylazine 3.5 mg/kg (Lloyd Pharmaceutical, Shenandoah, IA, USA, Cat # 139-236). Animals were placed on a heated platform (37 °C) to maintain body temperature during the test. ERGs were recorded from both eyes simultaneously using gold wire loops placed on each cornea, with a drop of methylcellulose on the corneal surface. A stainless-steel needle electrode (Rhythmlink, Columbia, SC, USA, Cat # RLSND110-1.0) was placed subdermally at the base of the tail as ground, and a stainless-steel needle electrode was placed subdermally in the ventral midline of the chin as the reference. Measurements were performed using an Espion e3 recording unit coupled to the ColorDome Ganzfeld LED stimulator (Diagnosys LLC, Lowell, MA, USA). The protocol included scotopic flash light intensities of 0.05, 0.5, and 5 cds/m^2^, a photopic flash with light intensity of 50 cds/m^2^ after 10 min of light adaptation, and 30 Hz photopic flicker at an intensity of 25 cds/m^2^ (background of 30 cds/m^2^). Oscillatory potentials were filtered using a bandpass filter set between 0.3 and 100 Hz. Results were analyzed in JMP 9.0 (SAS Americas, Cary, NC, USA) using an unpaired, two-tailed Student’s *t*-test.

### 4.6. Optokinetic Response (OR): Visual Acuity, Contrast Sensitivity

Visual acuity was assessed at 6 weeks post-STZ injection using an Optomotry testing apparatus (Cerebral Mechanics, Lethbridge, AB, Canada). Animals were dark-adapted briefly in a dim, red light-illuminated room. Each animal was then placed on an elevated platform inside the Optomotry apparatus, which uses four monitors to visually simulate a rotating cylinder of grated bars to approximate visual acuity testing. Using testing protocol established by Douglas et al. [29], rats were placed inside the Optomotry apparatus, and responses were measured for both clockwise and counterclockwise directions. Bars were set initially at 0.08 cycles/degree (c/d) and were either increased when tracking was observed or decreased when animals failed to track, in a stepwise fashion until tracking ceased. Tracking was defined as when the animal’s head would reflexively move at least 15° around the vertical in the same direction as the movement of the bars. Bars were centered on the animal’s head, then repositioned using a guide built into the software each time the animal moved its head. Animals were first tested with the bars drifting in the counterclockwise direction, then in the clockwise direction.

Contrast sensitivity was similarly measured at 6 weeks post-STZ injection using the same Optomotry testing apparatus following visual acuity testing. Instead of using a stepwise staircase progression of visual acuity levels, three defined visual acuity levels were selected: 0.18 c/d, 0.10 c/d, and 0.04 c/d. At each level, the percent contrast started at 100% between the black and white bars, then decreased in a stepwise fashion until no tracking was observed.

Both visual acuity and contract sensitivity results were analyzed in JMP (SAS Americas, Cary, NC, USA) using an unpaired, two-tailed Student’s *t*-test.

### 4.7. Histology

Rats were euthanized by CO_2_ asphyxiation at 8 weeks post STZ induction. Eyeballs were enucleated and fixed either in Davidson’s solution for paraffin embedding processes or in 0.1M sodium cacodylate-buffered 4% paraformaldehyde (pH 7.4) for cryo-embedding processes for 48 h at 4 °C. After fixation, samples underwent a cryoprotection procedure in 10% (for 1 h at room temperature), 20% (for 1 h at room temperature), and then 30% of sucrose solution (for 16 h at 4 °C) prior to embedding in either paraffin (Polysciences, Warrington, PA, USA, Cat # 27004) or O.C.T. Compound (Fisher Scientific, Houston, TX, USA, Cat # 23-730-571). For each eye, sagittal sections of 5 μm thickness (paraffin embedded samples) or 10 μm thickness (O.C.T. embedded-samples) were cut from nasal to lateral side of the globe. Every 5th slide was stained with hematoxylin and eosin (H&E, Poly Scientific, McKinney, TX, USA, Cat # S212A, S176). All stained slides were examined under a Nikon SMZ25 stereo microscope (Nikon, Tokyo, Japan), and selected slides were imaged using a Nikon Eclipse Ti-inverted research microscope (Nikon, Japan) for morphological evaluation of retinal architecture, including outer nuclear layer (ONL) thickness.

Antibodies (Table 1) against albumin and occludin were used to evaluate blood vessel integrity, as was Isolectin B4. Other antibodies against markers including Müller and astrocyte marker GFAP, neuron marker NeuN, PNA, mitochondria marker COX, synaptic markers Synaptophysin, CtbP2, apoptosis maker caspase-3, and stress marker TIAR were used to evaluate retinal condition. The marker E1A was used to label graft cells, and PEDF was used to demonstrate the protein secreted by graft.

### 4.8. Cell Culture

Rat RPCs (R28) were purchased from Kerafast (Boston, MA, USA, Cat # EUR201) and expanded in-house following the vendor’s instructions. In brief, two days prior to transplantation, cryopreserved rat RPCs were thawed and cultured in DMEM (Sigma-Aldrich, St Louis, MO, USA, Cat # D6046) supplemented with 10% calf serum (Sigma-Aldrich, St Louis, MO, USA, Cat # C8056), 1% MEM non-essential amino acids (Lonza, Walkersville, MD, USA, Cat # BW13-114E), 1% MEM vitamins (Lonza, Walkersville, MD, USA, Cat # BW13-607C), 1% GlutaMAX-I (Gibco, Gaithersburg, MD, USA, Cat # A12860), and 0.01% Gentamicin (Lonza, Walkersville, MD, USA, Cat # BW17-518Z), as described in the product sheet.

Human RPCs were thawed and cultured in fibronectin coated flasks in Advanced DMEM/F12 (Gibco, Gaithersburg, MD, USA, Cat # 12634) supplemented with 1% GlutaMAX supplement (Gibco, Gaithersburg, MD, USA, Cat # A12860), 1% N-2 Supplement (Gibco, Gaithersburg, MD, USA, Cat # A13707), 20 ng/mL EGF (Invitrogen, Waltham, MA, Cat # PHG0311), and 20 ng/mL FGF-basic (Invitrogen, Waltham, MA, USA, Cat # CTP0261) for 2 days for all experiments, with a medium change performed one day post-thaw. Conditioned medium was collected at the harvest for ELISA assay.

Cells for transplantation were harvested using TrypLE (Gibco, Gaithersburg, MD, USA, Cat # A12859) and formulated in BSS PLUS Irrigating solution (Alcon Laboratories, Fort Worth, TX, USA, Cat # 0065080050) at a concentration of 50K cells/µL for rat RPCs and 30K cells/µL for human RPCs. A dose of 2 µL cells per eye was administered for each injection. Following surgery, both cell number and viability were reassessed; cell counts were consistently within 20% of the intended dosing concentration, and post-transplantation viability remained above 85%.

### 4.9. ELISA

Collected conditioned medium at the harvest was used to measure hRPC PEDF secretion. PEDF levels were measured using the Human Serpin F1/PEDF DuoSet ELISA kit (R&D Systems, Minneapolis, MN, USA, Cat # DY1177-05) following the manufacturer’s instructions. Optical densities at 450 nm and 540 nm were measured on a BioTek Synergy HT microplate reader (Winooski, VT, USA). To create a standard curve, CurveExpert software 1.40 (Hyams Development) was used to generate the best-fit curve through the standard points. All samples were assayed in triplicate.

## 5. Conclusions

Diabetic retinopathy (DR) is a major cause of acquired visual dysfunction and blindness. It is a common condition that is rapidly increasing in prevalence, in association with underlying rising rates of obesity and type II diabetes within the global population. Current treatments for DR are dominated by intraocular anti-VEGF agents and steroids; however, no current therapy addresses the irreversible component of neuronal cell death within the retina that begins early in the disease process [30]. As a result, current treatments are of limited long-term effectiveness, and there remains a substantial unmet need for a therapy that will enhance the survival and function of retinal neurons in DR.

Here, we provide evidence that a cell-based treatment, (RPCs) developed for use in IRDs, may have applicability as a novel means of conferring cytoprotective effects in DR. These effects not only apply to neurons but to other impacted cell types within the retina as well. Of note, vascular integrity is greatly improved.

## Figures and Tables

**Table 1:** Antibodies used for immunohistochemistry.

Antibody	Manufacturer/Vendor	Catalog #
Albumin	Bethyl Labs (Montgomery, TX, USA)	A110-134A
Isolectin B4	Vector Labs (Newark, CA, USA)	B-1205
GFAP	DAKO (Glostrup, Denmark)	Z0334
Occludin	Thermo Fisher (Waltham, MA, USA)	33-1500
VEGF	Abcam (Cambridge, UK)	AB46154
Caspase 3	Cell Signaling (Danvers, MA, USA)	D175 (9661)
NeuN	Chemicon (Darmstadt, Germany)	MAB-377
TIAR	Cell Signaling	5137
COX (OxPhos)	Invitrogen (Waltham, MA, USA)	459600
Synaptophysin	DAKO	A-0010
PNA	Vector Labs	B1075
E1A (M73)	Abcam	ab140217
PEDF	Santa Cruz Biotechnology (Dallas, TX, USA)	SC-25594

## Data Availability

The original contributions presented in this study are included in the article. Further inquiries can be directed to the corresponding author.
